# Certainty of success: three critical parameters in coronavirus vaccine development

**DOI:** 10.1038/s41541-020-0193-6

**Published:** 2020-05-25

**Authors:** David C. Kaslow

**Affiliations:** PATH, 2201 Westlake Avenue, Suite 200, Seattle, WA 98121 USA

**Keywords:** Vaccines, Viral infection

## Abstract

Vaccines for 17 viral pathogens have been licensed for use in humans. Previously, two critical biological parameters of the pathogen and the host–pathogen interaction—*incubation period* and *broadly protective, relative immunogenicity*—were proposed to account for much of the past successes in vaccine development, and to be useful in estimating the “certainty of success” of developing an effective vaccine for viral pathogens for which a vaccine currently does not exist. In considering the “certainty of success” in development of human coronavirus vaccines, particularly SARS-CoV-2, a third, related critical parameter is proposed—*infectious inoculum intensity*, at an individual-level, and *force of infection*, at a population-level. Reducing the *infectious inoculum intensity* (and *force of infection*, at a population-level) is predicted to lengthen the incubation period, which in turn is predicted to reduce the severity of illness, and increase the opportunity for an *anamnestic response* upon exposure to the circulating virus. Similarly, successfully implementing individual- and population-based behaviors that reduce the *infectious inoculum intensity* and *force of infection*, respectively, while testing and deploying COVID-19 vaccines is predicted to increase the “certainty of success” of demonstrating vaccine efficacy and controlling SARS-CoV-2 infection, disease, death, and the pandemic itself.

## Introduction

In the absence of an existing, safe, effective vaccine for a pathogen (i.e., absent proof of clinical efficacy and safety), the risks and uncertainties in developing a new vaccine can be broadly divided into two categories: “biologic uncertainty”—the inherent biological ability of the candidate vaccine to elicit a protective immune response in humans with a safety profile that results in a positive benefit-risk balance; and, “execution uncertainties and risks”—the successful performance of literally thousands of tasks required to develop the vaccine. The subfactors that determine “biologic uncertainty” are perhaps the most difficult to overcome because most are largely immutable. Two dominant parameters that underlie “biologic uncertainty” are the safety/tolerability and the efficacy of a candidate vaccine. Unlike execution *risks*, such as the underlying failure rate of a production run under a given set of operational conditions, biologic *uncertainties* have a relatively binary outcome—the candidate vaccine does or does not have a favorable benefit-risk profile—that remains largely unchanged under a given set of epidemiologic conditions. Relatively because the outcome measures, such as efficacy and effectiveness, have inherent variability around the point estimate. This article revisits previously described key parameters of biologic feasibility proposed to determine the “certainty-of-success” (also referred to as the “probability of success”) in developing prophylactic vaccines^1^, but now in the context of coronaviruses.

Oftentimes safety and efficacy are inversely related, the so-called double-edged sword of attenuation^2^: an improvement in one resulting in a loss of performance in the other. Of the two, safety is often given priority over efficacy in regulatory review because the target populations of prophylactic vaccines are usually healthy. The risk tolerance for safety in the midst of an outbreak for a pathogen with a high *R*_0_ and a high case fatality rates may be higher^3^ than for vaccine use in routine immunization for relatively low-prevalence endemic diseases, particularly those with a low case fatality rate. Despite the paramount importance of safety, this article will focus on estimating the “certainty of success” from the efficacy/effectiveness side of the benefit-risk balance.

As noted above, the importance (and difficulty) of accurately estimating the performance of a candidate vaccine relative to the efficacy threshold in humans has been reviewed previously^1^. Herein the previously proposed paradigm that effective vaccines have been developed mainly for pathogens with lengthy incubation periods is re-explored as it pertains to active prophylactic immunization for coronaviruses, particularly SARS-CoV-2. Whereas in the original analysis, discussion of population-based effects of immunization (e.g., herd effects) were considered, here the focus is mainly at the level of the individual vaccinee, with the notable exception of the population-based effect of *force of infection* (see glossary of terms), and the continued circulation of virus in the population on the durability of individual immunity. Several simplifying assumptions are made, including that vaccinees are immunocompetent and share similar underlying condition profiles, that the kinetics of an effective acquired and anamnestic immune response is similar for different vaccine modalities, and that the immune responses elicited by vaccination prior to any previous pathogen exposure is at least similar to that acquired during natural infection.

## Analysis of biological feasibility of COVID-19 vaccine development

**Fig. 1:**
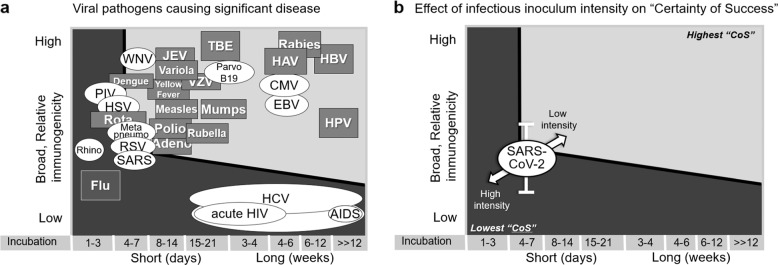
“Certainty of Success” of vaccine development as a function of *incubation period* and *broadly protective, relative immunogenicity*. **a** Two-dimensional analysis of 29 major human viral pathogens based on incubation period (*x*-axis; time, in days or weeks, from exposure to clinical signs or symptoms) and broad, relative immunogenicity (*y*-axis; high, moderate or low—see reference^1^ for definition). The 17 viral pathogens for which vaccine efficacy have been established are depicted in boxes with gray backgrounds; those for which vaccine efficacy has yet to be established are depicted in ellipses with white backgrounds. The area of graph associated with higher “certainty-of-success” for vaccine development (light gray) and lower “certainty-of-success” (dark gray) are separated by a thick black line. *(Reprinted from an open-access article licensed under a Creative Commons Attribution-NonCommercial 3.0 Unported License*^1^*.* **b** Effect of low and high infectious inoculum intensity on the assessment of the “certainty-of-success” (CoS) of SARS-CoV-2 vaccines. Interval bar (white) reflect the uncertainty in the inherent broadly protective, relative immunogenicity (see Glossary of Key Terms) associated with SARS-CoV-2 natural infection. Double-headed arrow (white with black outline) reflects the effect of infectious inoculum intensity higher (light gray) and lower (dark gray) “certainty-of-success” for vaccine development.

The previously proposed paradigm specifically considered, as part of the “certainty-of-success” analysis of biological feasibility, two critical properties of the many inherent biological properties of viral pathogens and the dynamic interaction with the human host: *incubation period*; and, *broadly protective, relative immunogenicity*. The combination of these properties appeared to have accounted for much of the successes so far achieved in vaccine development for viral pathogens (see Fig. 1a)^1^. In the original analysis conducted more than a dozen years ago, an effective, durable vaccine against SARS-CoV was predicted to be on the cusp of biological feasibility when applying *the 5-day incubation period rule*. In the current specific analysis for SARS-CoV-2 vaccine development, *infectious inoculum intensity* is elevated to a critical parameter because of its putative implications in assessing the “certainty of success” of developing an efficacious COVID-19 vaccine for use in an outbreak setting and in the design, conduct, and interpretation of COVID-19 vaccine efficacy and effectiveness studies.

### Incubation period and infectious inoculum intensity

*Incubation period*, defined as the time between exposure to the pathogen and onset of signs and/or symptoms of clinically apparent disease (see Box 1. Glossary of Key Terms), incorporates, in an empirically derived unit of time: (1) multiple inherent biological properties of the pathogen; (2) the dynamic interaction between the virus and the host; and (3) real world conditions of transmission (see *Force of infection* below). Factors that determine the incubation period include the amount of infectious virus in a typical inoculum, the infectivity of the viral pathogen, the rate of viral replication, the rate of viral clearance by a variety of host mechanisms, including innate and adaptive immunity, the impact of viral immune evasion tactics, and the viral load that results in signs or symptoms of disease. The clinical signs and/or symptoms that define the endpoint of the incubation period also impact the reported value. Not surprisingly, the incubation period can be quite variable, and retrospectively, difficult to precisely and accurately measure.

In its simplest iteration, the incubation period can be viewed as a race between: (1) the immune system’s ability to generate a sufficient and appropriate innate and/or adaptive response; and (2) the replication of the pathogen to a viral load that results in symptoms. As noted previously, an important inflection point occurs around 3 days when considering incubation periods for viral pathogens. The “certainty of success” for viruses, such as influenza (median incubation period 2 days, with a range of 1–4 days), that have short incubation periods do not benefit from the opportunity of an *anamnestic response* (see Box 1. Glossary of Key Terms). In addition, for vaccines against viral pathogens, particularly those with a short incubation period, protection against mild symptoms is often a much more difficult endpoint to achieve than protection against severe disease. In the absence of vaccine-induced, persistent, high-level immune effector function (e.g., circulating high-titer neutralizing antibodies and/or cytotoxic T-cell lymphocytes), early protection against lower level viral replication (i.e., early mild disease) may be more difficult to achieve than an *anamnestic response* (e.g., newly activated memory B- and T-cell responses) to protect against higher-level viral loads (i.e., more severe disease) that occur much later during infection. This model of being able to elicit high-level protection against severe disease, but not mild clinical symptoms (e.g., observed for rotavirus vaccines), will likely apply to SARS-CoV-2.

*Infectious inoculum intensity*, at an individual-level, and *force of infection*, at a population-level, are factors that may inversely contribute to the length of the *incubation period*, the *latent period*, and directly contribute the severity of symptoms associated with infection^4,5^. Equally, if not more relevant to “certainty of success” for vaccine development is the relationship between infecting dose and severity of disease^6^, as demonstrated by influenza inoculum dose-related rate of mild-to-moderate disease in a controlled human infection model^7^.

With respect to coronaviruses, an inverse correlation between the length of the incubation period and the severity of disease was recently evaluated from data collected during the 2003 SARS outbreak in Hong Kong. Comparing the length of the incubation period between fatal cases and non-fatal cases suggested a correlation between shorter incubation and greater severity, allowing for potential confounding by age, sex and occupation^8^. A similar finding was observed between the estimated incubation period of Middle East respiratory syndrome CoV (MERS-CoV) cases and mortality during the 2015 MERS outbreak in South Korea—patients who died had a shorter incubation period than patients who survived^9^.

With respect to SARS-CoV-2, Jiang et al.^10^ and Amodio et al.^11^ have both noted that a longer incubation time may lead to a high rate of asymptomatic and sub-clinical infection among immunocompetent individuals; however, an inverse relationship between incubation period and severity of disease has yet to be demonstrated. While the relationship between *incubation period* and perhaps more importantly, the *infectious inoculum intensity* of SRS-CoV-2 and severity of COVID-19 requires further validation, data consistent with an inverse relationship was highlighted by Sanche et al.^12^ when noting that a potential caveat of their estimation of a shorter incubation period [4.2 days (95% CI 3.5–5.1 days)] for SARS-CoV-2 than most other published reports is because most of their case reports were collected from the first few persons detected in each province, which may have biased case detection toward patients with more severe symptoms.

A noteworthy exception to the inverse relationship between incubation period and severity of disease comes from the observation that a longer incubation period among human influenza H7N9 cases was associated with a greater risk of death. Virlogeux et al.^13^ noted that H7N9 virus infection differs from H5N1, SARS, and MERS coronaviruses in several respects, including tropism limited to the human upper airways, the absence of a cytokine storm, and the stronger association of severe H7N9 disease with exacerbation of other underlying diseases, while H5N1, SARS, and MERS coronaviruses cause severe disease in otherwise healthy persons. As such, it is proposed here that the exception to the inverse relationship between incubation period and severity of disease is unlikely for SARS-CoV-2.

Admittedly confounded by multiple other factors, the population-based *force of infection* appears, in at least two recent examples, to be inversely associated with vaccine efficacy/effectiveness (VE/VEf). In the case of rotavirus VEf, a review of the first decade of post-licensure data from 24 countries showed a gradient of median VEf of 84%, 75%, and 57% in countries with low, medium, and high child mortality, respectively, for the monovalent vaccine based on a single human rotavirus strain, and a VEf difference of 90% and 45% in countries with low and high child mortality, respectively, for a pentavalent vaccine based on five bovine–human reassortant rotavirus strains^14^. Prelicensure rotavirus vaccine VE data demonstrate a similar gradient^15^, and, when analyzed by the pre-existing rotavirus disease burden (mortality)^16^ as an indicator of the *force of infection*, are consistent with an inverse association with VE, as suggested by Feiken, et al.^17^ This inverse association was also recently suggested during the regulatory evaluation of the malaria vaccine, RTS,S/AS01_E_. The European Medicines Agency (EMA) noted that “VE tends to be lower in high transmission areas”^18^ when analyzing the pivotal MAL-055 efficacy trial conducted in eleven research centers in seven sub-Saharan African countries, where the *force of infection* (categorized by annual mean *P. falciparum* parasite rate, age-standardized in 2 to 10-year olds^19^) differed by two orders of magnitude.

Lastly, this notion of an association between force of infection and severity of disease is consistent with multi-year observations of seroconversion rates for the four human endemic coronavirus and frequency of virus in hospitalized children^20^. Dijkman et al. reported that the frequency of infection observed via seroconversion in the study population, presumed to be associated with the relative force of infection in that population, had the same rank order of HCoV-OC43 ≥ HCoV-NL63 > HCoV-HKU1 ≥ HCoV-229E as the frequency of virus in hospitalized children, presumed to be associated with the severity of disease. Given the extensive spread of SARS-CoV-2, it should be possible to determine if a similar association between force of infection and severity of disease is observed during the current pandemic.

Box 1 Glossary of key terms Anamnestic response: A secondary or subsequent immune response that yields a faster, greater, and longer lasting immune response upon re-exposure to an immunogen than that induced during the preceding primary immune response.

Broadly protective, relative immunogenicity: A semi-quantitative term that captures the genetic diversity of the virus, and two aspects of the host immune responses: (1) the quality of the immune response that is elicited during and immediately after a primary infection; and (2) the ability and duration of that elicited immune response to protect against subsequent symptomatic reinfection.

Certainty of success: An estimate of the confidence in successfully demonstrating biological activity of a candidate vaccine based on pre-defined endpoints of efficacy/effectiveness.

Force of infection: Rate at which susceptible individuals in a *population* acquire an infectious disease in that *population.*

Incubation period: Time interval between infectious agent exposure moment in an *individual* and appearance of first sign or symptom of disease in that *individual.*

Infectious inoculum intensity: Magnitude (or area under the curve) in an *individual* of the infectious agent exposure at the exposure moment(s) associated with the duration of the incubation period in that *individual.*

Latent period: Time interval between infectious agent exposure moment in an *individual* and onset of period of infectious transmissibility to others in the *population*, which may be shorter or longer than the incubation period.

### Broadly protective, relative immunogenicity

A composite of additional biological features has been incorporated into a single semi-quantitative term, *broadly protective, relative immunogenicity* (see Box 1. Glossary of Key Terms; nominally categorized as high, moderate or low; also see Table 1), to provide a needed second dimension to refine the estimate of “certainty of success”. Specifically broadly protective, relative immunogenicity incorporates both genetic diversity of the virus, and two aspects of the host immune response: (1) the quality of the immune response that is elicited during and immediately after a primary infection; and (2) the ability and duration of that elicited immune response to protect against subsequent symptomatic reinfection. Factors relevant to coronaviruses, particularly SARS-CoV-2, that could contribute to categorizing *broadly protective, relative immunogenicity* might include the number of circulating coronavirus strains that cross-react or cross-protect against other coronaviruses^20^, the propensity for coronaviruses to propagate mutants during the incubation, latent, disease and recovery periods, the frequency of asymptomatic infections due to viral clearance by an adaptive immune response during the primary infection and during reinfection, and the durability of the protective immune response.

**Table 1:** Biological properties of influenzas and coronaviruses and virus–host interactions.

Viral pathogen	Incubation period (range)	Route of transmission	RNA genome	Size (kilobases)	Genetic stability	Immune response quality/duration	Broadly protective, rel. immunogenicity	References
Influenza H1-3	1–2 days (1–4 days)	DC/R	Segmented –ssRNA	13.6	Low	Mod/low	Low	^44–46^
Influenza H5	2–5 days (2–17 days)	DC/R		13.6	Low	Mod/low	Low	^47–54^
Influenza H7	5 days (1–10 days)	DC/R		13.6	Low	Mod/low	Low	^53,55–58^
hCoV-229E	3 days (2–5 days)	DC/R	α +ssRNA	27.3	Mod	Mod/low	Low	^59–62^
hCoV-NL63	*“2–4 days”*	DC/R		27.5	Mod	Mod/low	Low	^63–68^
SARS-CoV	4 days (2–15 days)	DC/R	β +ssRNA	29.7	Mod	High/mod	Mod	^8,23,69–78^
SARS-CoV-2	5 days (2–14 das)	DC/R/FO		29.9	Mod	TBD/TBD	Low↔Mod	^10,11,79–82^
hCoV-OC43	*“2–5 days”*	DC/R/FO		30.7	Mod	Mod/low	Low	^83–85^
hCoV-HKU1	*“2–4 days”*	DC/R		29.9	Mod	Mod/low	Low	^33,86–89^
MERS-CoV	5 days (2–15 days)	DC/R		30.1	Mod	Mod/low	Low	^9,38,40,41,89–95^

*Incubation period:* median (range); italicized are published but unconfirmed incubation periods.

*Route of transmission:* *DC* direct non-genital contact of secretions, *FO* fecal-oral, *R* respiratory droplets.

*RNA genome*: *+ssR* positive single-stranded RNA, *–ssR* negative single-stranded RNA, *α* alphacoronavirus, *β* betacoronavirus.

*Broadly protective, relative immunogenicity:* using a Delphi-type approach, incorporates in a single, semi-quantitative term [high; moderate (Mod); Low; or to be determined (TBD)]: (a) the genetic diversity of the virus (see column labeled “Genetic Stability”); and (b) two aspects of the host immune response: (1) the *quality* of the immune response that is elicited during and immediately after a primary infection (see first term in column labeled “Immune Response”); and (2) the ability and *duration* of that elicited immune response to protect against symptomatic reinfection (see second term in column labeled “Immune Response”).

As to genetic diversity, coronaviruses have the largest positive-sense, single-stranded RNA (+ss RNA) genomes known to cause disease in humans, from 27 up to 32 kilobases (kb), with the genus betacoronavirus SARS-CoV-2 at 29.9 kb (see Table 1). The long coronavirus genome displays a high degree of plasticity, particularly the Spike or S protein (see below), which can adapt with relative ease to exploit different cellular receptors, likely underlying the propensity of the four genera of animal coronaviruses to jump hosts^21^. While the bat- and rodent-derived alpha- and beta-coronaviruses have likely attempted to jump into humans frequently, three cross-species transmission events, over the past dozen and a half years, have resulted in outbreaks of SARS-CoV, MERS-CoV, and SARS-CoV-2 in the human population. Four well adapted “common cold”-type coronaviruses also widely circulate in humans (i.e., hCoV-229E, -NL63, -OC43, and -HKU1)^22^. Of the nine open-reading frames encoded in the coronavirus genome and the four structural proteins, the Spike or S protein, particularly the receptor-binding domain (RBD) in the S1 subunit and the S2 subunit, is of specific interest because this essential structural protein, expressed in multiple copies on the lipid bilayer envelope of the virus, determines in part the host range through its role in host cell attachment, fusion, and entry^23–26^. In the case of SARS-CoV-2, evolution within the modular structure of the S protein, the main target of protective immunity, may be driven by the known error rates of coronavirus RNA-dependent RNA polymerases, and the marked capacity of coronaviruses to employ homologous recombination in the context of coinfections. While not nearly as genetically diverse as the +ss RNA hepatitis C virus nor as stable as the +ss RNA hepatitis A virus, SARS-CoV-2 is likely susceptible to moderate genetic diversity, despite the SARS-CoV-2 RBD already significantly higher binding affinity to the human angiotensin-converting enzyme 2 (ACE2) receptor than SARS-CoV RBD^27^. So, although SARS-CoV-2 outbreak appeared after SARS-CoV, phylogenetically, SARS-CoV-2 appears to be an “older” virus more closely related to the progenitor bat CoV than SARS-CoV^26^.

One surrogate for the level of *broadly protective, relative immunogenicity* is the frequency of reinfection. Reinfection with the four circulating human “common cold”-type coronaviruses appears to be a frequent event. Examples from the two human coronaviruses studied since the 1960s, include a 4-year study of hCoV-OC43 infection in Tecumseh, Michigan following an hCoV-229E outbreak in 34% of the study population^28^. The incidence of hCoV-OC43 infection in children <5 years of age was high, yet subsequent symptomatic reinfection, albeit mild except chronic bronchitis in some, was quite frequent in older children and in adults. When analyzed immunologically, >80% of these subsequent symptomatic infections occurred despite prior neutralizing antibodies, calling into question the protective value of circulating neutralizing antibody (or the assays used)^29^. Reinfections were also frequently observed, commonly associated with respiratory symptoms, for these two human coronaviruses in young children^30^, as well as in a longitudinal study of working adults^31^. Similar but less robust epidemiological findings have been reported from the more recently described endemic human coronaviruses, hCoV-NL63 (first described in 2004^32^) and hCoV-HKU1 (first described in 2005^33^) (see also Table 1 for references).

Controlled Human Infection Model (CHIM) studies^34^ provide another source of evidence to inform categorization of *broadly protective, relative immunogenicity*. In the case of hCoV-229E, CHIM studies in adults document susceptibility to symptomatic reinfection despite the presence of detectable antibodies, although homologous re-challenge a year later led to only asymptomatic reinfection^35^. As noted by Callow et al.^35^ the human challenge data are consistent with the notion that adults have human coronavirus infections on a 2–3 year cyclic pattern and that “protective amounts of antibody may have disappeared by 2 years, and that if we had been able to reinoculate the volunteers after a further year, the reinfection rate would have been even higher”. The totality of the findings from natural and controlled challenge infections, in conjunction with a moderate degree of genetic diversity in these four endemic human coronaviruses, led to a “Low” *broadly protective, relative immunogenicity* categorization in Table 1.

Similar to the four endemic coronaviruses, the quality and the durability of the protective immune response after natural infection with the three human epidemic coronaviruses appear to be “Low” or at best “Moderate”, the difference being that severe disease has been observed more frequently for SARS-CoV, MERS-CoV, and SARS-CoV-2 than the common cold human coronaviruses. Several longitudinal sero-epidemiology studies after the SARS-CoV outbreak reported a high post-convalescent seroconversion rate with IgG peaking at 2–4 months in patient serum samples. In a small sample size study evaluating neutralizing activity in serial serum samples from patients with SARS, >85% contained neutralizing antibodies (NAb) against SARS-CoV and most of the NAb activities could be attributed to immunoglobulin G (IgG)^36^. However, the duration of circulating IgG appeared relatively short-lived as the longest longitudinal study reported that at 3 years, the IgG positivity had declined to 55.56%^37^. If the incubation period of SARS-CoV is sufficiently long to allow a protective *anamnestic response*, then subsequent re-exposure would likely lead to an asymptomatic infection. Similar seroconversion and NAb rates have been published for MERS-CoV, with several studies suggesting that antibody levels and longevity following MERS-CoV infection are correlated with disease severity^38–40^. Okba et al.^41^ reported that all fifteen severe MERS-CoV cases tested positive in all tested platforms up to 1 year after disease onset, indicating a robust immune response of high antibody titers in severe cases; however, low or undetectable seroconversion rates and undetectable neutralizing antibodies were observed after most asymptomatic and some mild MERS-CoV infections. Early data from the current SARS-CoV-2 pandemic suggest a similar pattern of immune responses in severe and mildly symptomatic SARS-CoV-2 patients^42^. Given these data, it is tempting to speculate that a lower *infectious inoculum intensity* leads not only to a longer *incubation period* and less severe disease, but also to a less robust *broadly protective, relative immunogenicity* after natural infection. Active immunization that optimally balances efficacy with reactogenicity/tolerability may represent the best of both worlds—robust broadly protective, relative immunogenicity without the severity of disease by administration of a high inoculum intensity without infectiousness.

While it is too soon to have significant empiric data on the durability of SARS-CoV-2 immune responses to protect against symptomatic reinfection, the durability after natural infection as well as the long-term efficacy of active immunization may be influenced by the extent to which SARS-CoV-2 and/or related cross-reacting human coronaviruses continue to circulate in the population or “herd”. Under conditions in which insufficient herd immunity exists to curtail or even eliminate circulation of these viruses in the “herd”, repeated sub-clinical infections may serve to maintain a protective immune response in an individual of the “herd”. As the prevalence of these viruses diminishes in the “herd” as a result of adequate vaccine coverage and/or naturally acquired immunity, likely so will durability of protective immunity in the individuals in the “herd” as subsequent sub-clinical infections no longer occur and no longer serve to naturally “boost” an adequate protective immune response. In such situations where circulation of relevant coronaviruses significantly diminish, re-vaccination must be considered if a high “certainty-of-success” for long-term protection against future outbreaks is sought.

A limitation of this rudimentary approach taken herein to assign a qualitative value to this composite biological feature of pathogens (Fig. 1a) and SARS-CoV-2 (Fig. 1b) is that it did not employ nor benefit from more powerful tools such as system biology analyses or mathematical models, which have been shown to provide important non-intuitive insights into host–virus interactions. Such tools would need to be brought to bear on this topic for a more rigorous evaluation and for a more accurate and precise placement in the broad categories of high, moderate, and low levels of *broadly protective, relative immunogenicity* depicted in Fig. 1a, b.

## Estimating certainty-of-success

By simultaneously considering the incubation timeline with the genetic diversity and the quality and durability of the host immune response, an approach to estimating the “certainty of success” based on biological feasibility emerges (Fig. 1)^1^. The model predicts an inverse relationship between the length of the *incubation period* and the level of the *broadly protective, relative immunogenicity* needed to achieve an equivalent “certainty of success”. That is, for those pathogens that have a short incubation period and less opportunity for protection through an *anamnestic response*, a higher *broadly protective, relative immunogenicity* is needed to have a high “certainty-of-success”; likewise, for those pathogens having a long *incubation period* that benefit from protection through an *anamnestic response*, a lower *broadly protective, relative immunogenicity* is needed to have a high “certainty-of- success”.

The association between *incubation period* and “certainty-of-success” is just that—an association. Although the proposed model may well accommodate the dataset presented in Fig. 1a, cause and effect clearly has not been demonstrated. In fact, many of the viral pathogens that have short incubation periods also cause hit-and-run, local mucosal infections. These pathogens (e.g., Rhinovirus, Influenza, RSV, PIV, and MPV) cause relatively brief illnesses and have limited tropism. Whether the latter is the key parameter of biologic feasibility that determines “certainty-of-success” for developing prophylactic vaccines for these pathogens remains to be determined. As described in detail previously^1^, given its short *incubation period* and low *broadly protective, relative immunogenicity* (see Table 1), influenza is a particular outlier in estimating “certainty of success” because of a relatively predictable transmission season, and the availability of annual immunization with an updated vaccine just prior to exposure in high-resource settings. In many low-resource settings, the latter is not an option and a fit-for-purpose influenza vaccine that does not require annual updating and annual administration has yet to be developed.

### Predictions for the biological feasibility of developing effective COVID-19 vaccines

In the end, “predictions ought to count more than accommodations, because of the risk of ‘fudging’ that accommodations run and predictions avoid^43^”. With this mind, four guiding principles and three implications on the design, conduct and interpretation of vaccine clinical trials (see Box 2) are offered for pressure-testing the three factors identified herein (see Fig. 1b), as SARS-CoV-2 candidate vaccines advance into proof-of-efficacy studies. While many other factors that are not easily controlled will affect the robustness of the principles and implications proposed, some consideration to the factors under human control would seem prudent. The one factor that emerges for consideration in SARS-CoV-2 vaccine development and implementation is reducing the *infectious inoculum intensity* (and *force of infection*, at a population-level) to lengthen the incubation period, reduce the severity of illness, and increase the opportunity for an *anamnestic response* upon exposure to the circulating virus. Successfully implementing individual- and population-based behaviors that reduce the *infectious inoculum intensity* and *force of infection*, respectively, while testing and deploying COVID-19 vaccines may be a critical human-controlled factor in assuring the “certainty of success” through immunization in controlling and eliminating SARS-CoV-2 infection, disease, death, and the pandemic itself.

Box 2 Proposed guiding principles and implications for COVID-19 vaccine clinical trials **Proposed guiding principles in determining “certainty of success”**

1. Reducing the infectious inoculum intensity will:
  - lengthen the incubation period.
  - lengthen the latent period.
  - increase vaccine efficacy.
2. Lengthening the incubation period (see “Note” below) will:
  - reduce the risk of severe disease.
  - increase the opportunity for anamnestic responses upon subsequent infectious inoculum exposure.
3. Lengthening the latent period will:
  - increase the herd effect of naturally acquired immunity and/or vaccine-induced protective immunity.
4. Increasing the opportunity for anamnestic responses will:
  - increase vaccine efficacy beyond that predicted by Circulating antibody levels.
  - increase durability of protective immunity while the pathogen still circulates in the population.
  **Implication for vaccine trial design, conduct, and interpretation:**
5. Assessing/estimating the incubation period during vaccine efficacy trials could provide insights into the infectious inoculum intensity and could provide insights into benefit-risk assessments for different use cases (e.g., high-risk first responders and healthcare workers with high-level exposure vs. general use to protect against low-level exposure during reopening after mitigation vs. routine use during interpandemic period).
6. Determining vaccine efficacy for specific use cases and comparing vaccine efficacy of different vaccines should account for the infectious inoculum intensity in that specific use case setting and in the vaccine efficacy trial setting, respectively.
7. Evaluating the correlates of protection (particularly in infectious inoculum intensity settings in which the incubation period is ≥5 days) should include measures of anamnestic responses, in addition to circulating functional antibody levels.

**Note:** By reducing the infectious inoculum intensity [through individual measures (e.g., handwashing, other hygiene practices, and personal protective equipment) and/or population-based measures to reduce the force of infection] or by naturally acquired or vaccine-induced protective immunity.
